# Mucolytic Drugs Ambroxol and Bromhexine: Transformation under Aqueous Chlorination Conditions

**DOI:** 10.3390/ijms25105214

**Published:** 2024-05-10

**Authors:** Sergey A. Sypalov, Ilya S. Varsegov, Nikolay V. Ulyanovskii, Albert T. Lebedev, Dmitry S. Kosyakov

**Affiliations:** Laboratory of Environmental Analytical Chemistry, Core Facility Center “Arktika”, M.V. Lomonosov Northern (Arctic) Federal University, Northern Dvina Emb. 17, 163002 Arkhangelsk, Russia; s.sipalov@narfu.ru (S.A.S.); i.varsegov@narfu.ru (I.S.V.); mocehops@yandex.ru (A.T.L.); d.kosyakov@narfu.ru (D.S.K.)

**Keywords:** ambroxol, bromhexine, chlorination, disinfection byproducts, chromatography, high-resolution mass spectrometry

## Abstract

Bromhexine and ambroxol are among the mucolytic drugs most widely used to treat acute and chronic respiratory diseases. Entering the municipal wastewater and undergoing transformations during disinfection with active chlorine, these compounds can produce nitrogen- and bromine-containing disinfection by-products (DBPs) that are dangerous for aquatic ecosystems. In the present study, primary and deep degradation products of ambroxol and bromhexine obtained in model aquatic chlorination experiments were studied via the combination of high-performance liquid and gas chromatography with high-resolution mass spectrometry. It was shown that at the initial stages, the reactions of cyclization, hydroxylation, chlorination, electrophilic *ipso*-substitution of bromine atoms with chlorine, and oxidative N-dealkylation occur. Along with known metabolites, a number of novel primary DBPs were tentatively identified based on their elemental compositions and tandem mass spectra. Deep degradation of bromhexine and ambroxol gives twenty-four identified volatile and semi-volatile compounds of six classes, among which trihalomethanes account for more than 50%. The specific class of bromhexine- and ambroxol-related DBPs are bromine-containing haloanilines. Seven of them, including methoxy derivatives, were first discovered in the present study. One more novel class of DBPs associated with bromhexine and ambroxol is represented by halogenated indazoles formed through dealkylation of the primary transformation products containing pyrazoline or tetrahydropyrimidine cycle in their structure.

## 1. Introduction

**Figure 1:**
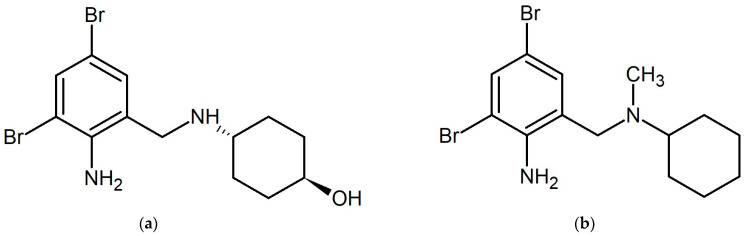
The structural formulas of ambroxol (**a**) and bromhexine (**b**).

The release of a variety of bioactive pharmaceuticals into municipal wastewater poses a serious problem for aquatic ecosystems due to the low efficiency of water-treatment facilities in relation to such micropollutants. The situation was worsened significantly due to the coronavirus (COVID-19) pandemic and the associated uncontrolled increase in the consumption of some types of pharmaceuticals [1,2,3]. In addition to antiviral drugs (remdesivir, aviptadil, umifenovir, etc.) approved for the treatment and prevention of coronavirus infection [4], mucolytic agents have also been widely used to relieve symptoms caused by excess mucus in the respiratory tract and suppress coughing. Among them ambroxol (AMB, 4-[(2-amino-3,5-dibromobenzyl)amino]cyclohexanol, CAS 107814-37-9, Figure 1a) and bromhexine (BRO, 2-amino-3,5-dibromo-N-cyclohexyl-N-methylbenzylamine, CAS 3572-43-8, Figure 1b) have found the greatest use in various countries. Along with mucolytic action, these compounds also showed direct effectiveness in inhibiting viral proteins [5].

According to the current recommendations [6,7,8], the therapeutic doses of AMB and BRO for adults are up to 90 and 32 mg/day, respectively, with 10–15% of the ingested drug being excreted unchanged from the body. It is important to note that during metabolism, bromhexine undergoes N-dealkylation and hydroxylation, with the formation of ambroxol as the main metabolite [9]. Thus, the use of these mucolytics inevitably leads to the release of AMB and BRO into wastewater and subsequently into water-treatment plants. The available literature data [10,11] confirm the presence of AMB in wastewater (from ng L^−1^ to μg L^−1^) with maximum concentrations in the winter months, which is explained by an increase in the frequency of seasonal respiratory diseases.

Like other pharmaceutical drugs, mucolytics can undergo chemical transformations at various stages of wastewater treatment. This especially applies to disinfection processes, in which chlorine-containing reagents are most widely used [12,13]. The advantages of such disinfectants based on the combination of their high efficiency, low cost and availability, are largely offset by the associated problems. The main issue is the formation of disinfection byproducts (DBPs) [14,15,16] as a result of interactions between active chlorine and dissolved organic matter. Most of them have carcinogenic, mutagenic and other adverse effects on organisms [17] and are often significantly more dangerous than the parent compounds [18,19]. Among the variety of the known DBPs (>800 compounds) [20], nitrogen-containing disinfection byproducts (N-DBPs) deserve special attention due to their increased toxicity [21].

AMB and BRO are no exception and, when interacting with active chlorine, they can form not only well-known trihalomethanes (THMs) but also haloacetonitriles (HANs) [8] and hazardous bromine-containing DBPs (Br-DBPs). Moreover, in a recent study on the aqueous chlorination of mucolytics a novel class of N-DBPs, namely, halogenated anilines (2,4,6-tribromoaniline and 2-chloro-4,6-dibromoaniline) [8], was discovered. The detection of Br-THMs and Br-HANs, as well as tribromoaniline, indicates the participation of bromine atoms of AMB and BRO in the reactions and their influence on the spectrum of the resulting products. This fact was confirmed to some extent by the study of the chlorination processes of a number of other bromine-containing compounds, such as antiviral drugs and pesticides [22,23]. It is worth noting that Br-DBPs are typically 1–2 orders of magnitude more toxic than chlorine-containing analogues [17,24].

Despite the widespread use of AMB and BRO, their degradation under water disinfection conditions is still poorly studied. The available literature contains only the recent study by Wang et al. [8], which was focused on the volatile DBPs and used gas chromatography-mass spectrometry for their identification. This approach significantly limits the ability to detect minor deep degradation products, and especially nonvolatile and thermolabile metabolites [25,26] formed at the early stages of AMB and BRO transformations. At the same time, these primary products may be more toxic to mammals than volatile DBPs [27], which necessitates the need to control their formation.

In this regard, the combinations of both gas and liquid chromatography with high-resolution mass spectrometry (GC-HRMS and HPLC-HRMS) have significant advantages in the comprehensive characterization of the chemical composition of DBPs. This approach has successfully proven itself in the study of processes occurring during the disinfection of drinking water and wastewater, as well as swimming pool water [22,28,29,30,31,32,33,34]. Thus, the purpose of this study was to reveal main degradation pathways and characterize in detail both the primary (intermediate) and final disinfection byproducts formed under the conditions of aqueous chlorination of bromhexine and ambroxol.

## 2. Results and Discussions

### 2.1. Primary Transformations of Bromhexine

**Figure 2:**
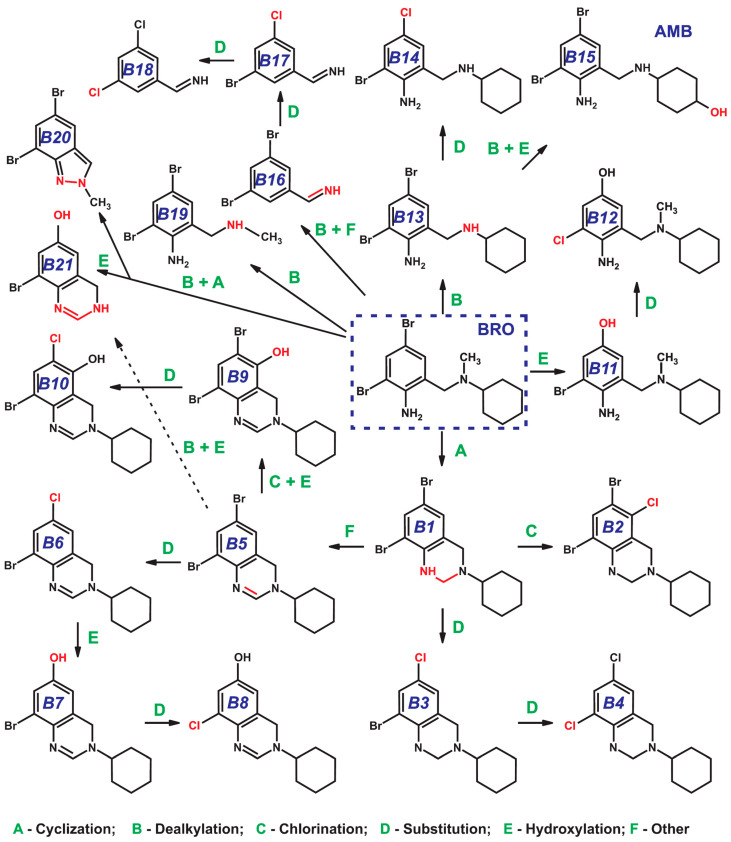
Bromhexine degradation pathways and primary transformation products formed during the aqueous chlorination.

The study of model reaction mixtures of bromhexine with active chlorine (sodium hypochlorite) via HPLC-HRMS with electrospray ionization (ESI) revealed at least 21 disinfection byproducts (Table 1) belonging to the class of N-DBPs. Based on the calculated elemental compositions and the obtained tandem mass spectra, the chemical structures for each detected compound were tentatively identified and the routes for their formation were proposed (Figure 2).

One of the key processes occurring during BRO transformation is cyclization, which is caused by the formation of a covalent bond between aromatic amino group and methyl substituent in tertiary amine moiety. This results in the formation of the product **B1** with an additional tetrahydropyrimidine cycle in its structure. This compound is already known as BRO metabolite formed in animals and humans and described in a number of literature sources [35,36]. The cyclization mechanism may involve electrophilic substitution of the hydrogen atom in the aromatic NH_2_ group with chlorine and further interaction of the resulting chloramine moiety with the reactive methyl group in aliphatic tertiary amine accompanied by the elimination of HCl and formation of the C-N bond. The reaction is promoted by the formation of an energetically favorable six-membered ring.

Regardless of the cyclization mechanism, product **B1** is an important intermediate in the formation of at least half of the detected N-DBPs. Thus, its further transformations involve introduction of a chlorine atom into the aromatic ring (chlorination, **B2**) and the substitution of one or two bromine atoms with chlorine (**B3** or **B4**, respectively). As was recently demonstrated [37], the latter process proceeds via an electrophilic *ipso*-substitution (*ipso*-S_E_) mechanism promoted by the presence of the substituents with high electron donating ability (-NH_2_ and -OH) and leads to the elimination of bromine cation. Being a highly reactive species, Br^+^ may attack various organic substrates available in the solution, contributing to further increase in the number of Br-DBPs. In particular, this explains the formation of such compounds as 2,4,6-tribromoaniline which was detected among BRO degradation products by Wang et al. [8] and in the present study (Section 2.4). Alternatively, Br^+^ may be further oxidized to bromate-anions (BrO_3_^−^) possessing pronounced carcinogenic properties [38].

The result of electrophilic attack of active chlorine on the aromatic secondary amino group in **B1** is the formation of a double bond (N=C) in the tetrahydropyrimidine cycle (**B5**). Obviously, this reaction proceeds through the formation of unstable chloramine (S_E_ mechanism) and subsequent elimination of HCl. This process is energetically favorable due to the increase in the conjugation system and proceeds quite easily, yielding a wide range of primary products with double bond (**B6**–**B10**, **B21**) formed mainly as a result of **B5** transformations. As in the case of **B1**, they involve *ipso*-substitution of Br with Cl (**B6**, **B8**, **B10**) and hydroxylation resulting in the replacement of a bromine or chlorine atom with a hydroxyl group (**B7**, **B9**). Since nucleophilic substitution cannot easily occur under rather mild conditions of aqueous chlorination, radical processes may play a key role in the reaction mechanism, especially those involving hydroxyl radicals generated in the presence of active chlorine. The parent bromhexine molecule is also capable of hydroxylation (**B11**) and *ipso*-substitution reactions (**B12**); however, in this case, only two intermediate products were detected.

Along with cyclization, the most important direction of BRO transformation is N-dealkylation. The possibility of such transformations as a result of one-electron transfer was confirmed in a recent study [39]. In the case of bromhexine, the loss of the methyl or cyclohexyl substituents, as well as their consecutive elimination, can occur. Demethylation leads to the occurrence of an intermediate **B13**, which can further undergo *ipso*-substitution of bromine for chlorine (**B14**) and hydroxylation with the formation of ambroxol (**B15**)—a known metabolite of BRO in living organisms [35,36]. The loss of cyclohexane moiety, in turn, is accompanied by cyclization with the formation of a new six-membered (C-N bond, **B21**) or five-membered (N-N bond, **B20**) ring. In addition, the BRO dealkylation product (**B19**) that does not contain a heterocycle in its structure is also observed in the reaction mixture. This confirms the key role of the methyl and amino groups of bromhexine in the formation of a wide range of disinfection byproducts. As in the previous case, the loss of two alkyl substituents is accompanied with the elimination of the -NH_2_ group. The resulting intermediate (**B16**) undergoes sequential ipso-substitution of bromine atoms with chlorine (**B17**, **B18**).

### 2.2. Primary Transformations of Ambroxol

Unlike BRO, AMB gives a relatively narrow range of primary transformation products. Apparently, this is due to the absence of a methyl substituent at nitrogen atom, which plays a key role in cyclization reactions. In total, only six compounds with different elemental compositions were detected in the reaction mixture via HPLC-HRMS (Table 2). In some cases, isomeric compounds with close retention times were observed on the chromatogram. Most likely they were positional isomers (introduction of substituents at different positions in aromatic ring); thus, only the dominant ones are included in Table 2.

**Figure 3:**
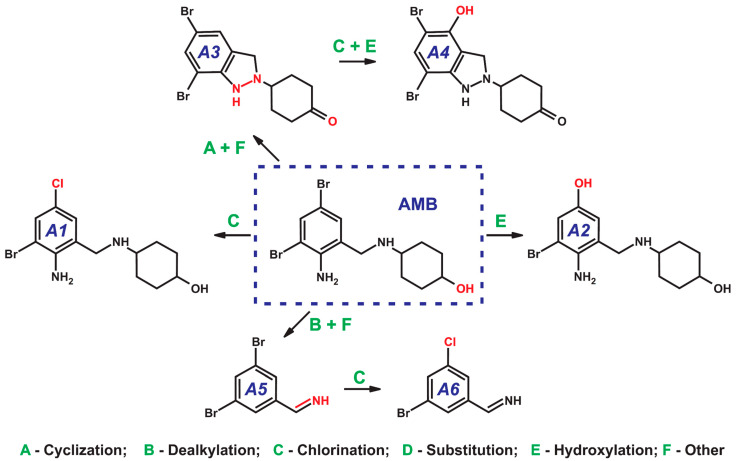
Scheme of the formation of detected DBPs during the interaction of ambroxol with active chlorine.

As in the case of BRO, the proposed scheme of AMB transformations (Figure 3) involves substitution of a bromine atom with chlorine (chlorination via *ipso*-S_E_ mechanism, **A1**) or a hydroxyl group (radical hydroxylation, **A2**). The latter compound is already known as a product resulting from aquatic photolysis of the pharmaceutical drug [40] and this fact can be considered as an additional proof of the assumed radical hydroxylation mechanism under the action of active chlorine.

Cyclization of AMB is possible only through the formation of a five-membered pyrazoline ring with an N-N bond. This process is accompanied by the oxidation of the hydroxyl group in cyclohexane moiety to a ketone (**A3**). This is confirmed by the observed elimination of CO in its tandem mass spectrum. The further transformation of **A3** proceeds mainly through radical hydroxylation and results in the formation of **A4**.

Oxidative dealkylation of AMB with elimination of cyclohexane moiety leads to the formation of the same product as in the case of BRO (**A5** = **B16**). Subsequently, this intermediate is subjected to an *ipso*-substitution of bromine by chlorine (**A6** = **B17**). However, it was not possible to detect the further transformation product with two chlorine substituents in the aromatic ring observed in the experiments with BRO (**B18**).

### 2.3. Transformation Dynamics and Effect of Active Chlorine Dosage

As shown in Figure 2 and Figure 3, BRO and AMB primary transformations proceed in different pathways and through the formation of a number of intermediate products. Thus, their concentrations and ratios in the reaction mixtures may vary significantly depending on the reaction time and active chlorine dosage. In our experiments chromatographic peak areas of parent compounds and the detected transformation products were monitored over 50 h at two active chlorine levels (4 and 7 mg L^−1^) close to actual concentrations created during water disinfection and corresponding to ~2-fold and ~4-fold molar excess towards substrates, respectively.

**Figure 4:**
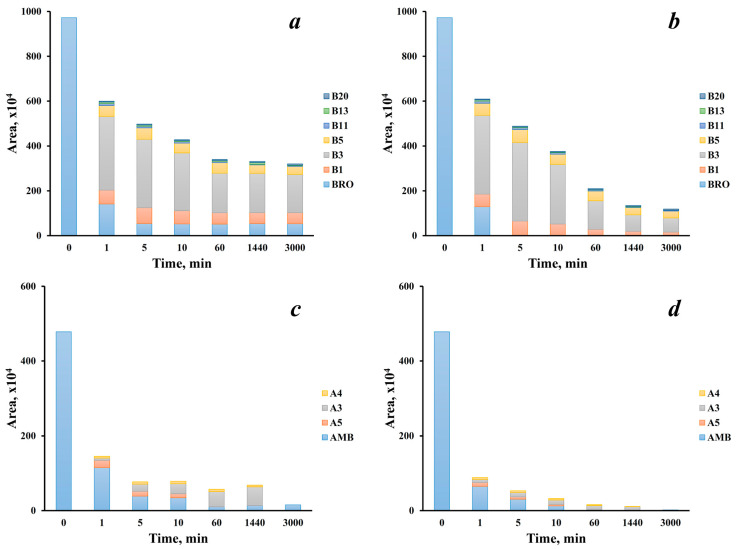
Chromatographic peak areas of parent compounds and primary transformation products of bromhexine (**a**,**b**) and ambroxol (**c**,**d**) at active chlorine concentrations of 4 (**a**,**c**) and 7 (**b**,**d**) mg L^−1^ depending on the reaction time.

The obtained results (Figure 4) demonstrate high reactivity of both drugs in interactions with the chlorinating agent—the concentrations of BRO and AMB rapidly decrease during first 5 min reaching values that are an order of magnitude lower than the initial ones.

Due to easier cyclization, BRO is more susceptible than AMB to the action of active chlorine. Thus, in the presence of a sufficient excess of chlorine, this compound is not detectable from the fifth minute of the reaction, while residual AMB can be found even in the sample taken 1 h after adding the hypochlorite solution (Figure 4b,d). Moreover, BRO differs with much higher ratios of primary degradation products and parent compound. The former are represented mainly by the first cyclization product B1 and its derivatives B3 and B5. Concentrations of all these primary intermediates increase sharply at the beginning of the reaction, reach the maximum in the range of 1–5 min and then gradually decrease due to conversion to the final products (Figure 4b). It is worth noting that compound B3, being the product of bromine substitution with chlorine, predominates in the reaction mixture throughout the entire studied period of time. This surprising fact indicates the unexpected ease of *ipso*-substitution reactions under the conditions of aquatic chlorination. The same pattern is observed at the lower chlorinating agent dosage (Figure 4a). However, in this case concentrations of all presented compounds remain almost constant after 60 min due to the lack of active chlorine.

A different picture is typical for the dynamics of AMB transformation. Unlike BRO, at the lack of active chlorine this compound rapidly transforms to the intermediate A5 (elimination of cyclohexane moiety) and other deep degradation products (Figure 4c). At the same time, concentration of the cyclization product A3 gradually increases over 24 h which makes this metabolite dominate among primary degradation products. However, after the next 26 h the latter were not found in the reaction mixture. With an excess of active chlorine (Figure 4d), this pattern persists, but the maximum concentration shifts to the reaction time of 10 min.

### 2.4. Deep Degradation Products

To reveal deep degradation products of BRO and AMB, the reaction mixtures with a large excess (10:1 *w*/*w*) of active chlorine relative to the substrates were prepared and kept for 30 min until the reaction was complete. The choice of process duration was determined by the results of studying the chlorination dynamics (Figure 4) showing the completion of the active stage of transformation 10–60 min after mixing the reagents even at low dosages of active chlorine. Analysis by gas chromatography–high-resolution mass spectrometry with headspace solid-phase microextraction (HS-SPME-GC-HRMS) enabled detection of twenty-four volatile and semi-volatile DBPs (Table 3). These compounds belong to six classes: trihalomethanes (THMs), dihaloacetonitriles (DHANs), haloacetic acid esters (HAEs), haloanilines, halobenzenes and haloindazoles.

As expected, the main deep degradation products were trihalomethanes, which account for approximately 90% of all detected compounds (based on the chromatographic peak area ratios) and are known as final products of chlorination of the dissolved organic matter. Their formation from AMB and BRO during aquatic chlorination has already been demonstrated in [8]. The results of the present study (Table 3) show that under identical conditions and a large excess of active chlorine, BRO and AMB differ only by the amount of only one product of this class. Despite the dominance of dibromochloromethane for both drugs (>50% of all volatile DBPs), BRO is characterized by the promoted formation of bromodichloromethane (23% of all volatile DBPs) while AMB gives half as much of this product.

In turn, AMB produces 6 times more dihaloacetonitriles, which is also consistent with the data from [8]. In addition, the content of methylated haloacetic acids in AMB degradation products is almost 15 times higher than that of BRO. It should be noted that DHANs and HAEs are also typical products of deep degradation of organic substances when interacting with active chlorine.

Semi-volatile DBPs specific to the studied pharmaceuticals are haloanilines. Wang et al. [8] were the first to establish the possibility of the formation of 2,4,6-tribromoaniline and 2-chloro-4,6-dibromoaniline from BRO and AMB. However, our results obtained with HS-SPME-GC-HRMS indicate the possibility of the formation of a much wider range of products of this class. Thus, nine additional haloanilines were detected and tentatively identified in the reaction mixtures. Among them, the products of the abovementioned bromine *ipso*-substitution with chlorine predominate, including fully substituted 2,4,6-trichloroaniline being the main representative of haloanilines in the BRO-derived DBPs. Special attention should be drawn to halomethoxyanilines, whose two representatives were found for the first time. Their formation may be caused by methylation of the corresponding hydroxylated compounds whose precursors were detected via HPLC-HRMS (Table 1 and Table 2). In this case, small amounts of methanol used as a solvent for BRO and AMB could act as a methylating agent and the formed products were preferentially extracted by HS-SPME due to their higher volatility compared to the parent hydroxylated DBPs. It should be noted that the transformation of BRO is characterized by a three-fold predominance of haloanilines in the reaction mixture compared to that of AMB.

Deeper degradation of haloanilines results in the elimination of amino group and formation of halobenzenes. One representative of this class, 1,3-dibromo-5-chlorobenzene, related to the abovementioned 2,4-dibromo-6-chloroaniline, was first discovered in trace amounts among both BRO and AMB DBPs.

One more group of the novel DBPs associated with BRO and AMB degradation is represented by halogenated indazoles. Their formation proceeds through dealkylation of the primary transformation products containing pyrazoline or tetrahydropyrimidine cycle in their structure. Even though only two such compounds (5-bromo-7-chloro-2-methylindazole and 5,7-dibromo-2-methylindazole) were found in small amounts, their discovery is of interest due to the high reactivity of the N-N bond and clearly confirms the occurrence of cyclization processes at the initial stages of aqueous chlorination.

## 3. Materials and Methods

### 3.1. Chemicals and Reagents

Commercially available preparations of ambroxol hydrochloride (≥98.0%) and bromhexine hydrochloride (≥98.0%) were purchased from Sigma-Aldrich (Darmstadt, Germany). Formic acid (ACS reagent, ≥96%), sodium chloride (ACS reagent, ≥99.0%) and sodium phosphate dibasic (ACS reagent, ≥99.0%) obtained from Sigma-Aldrich (Darmstadt, Germany), *ortho*-phosphoric acid (≥87%, Komponent-Reaktiv, Moscow, Russia), methanol (for gradient HPLC, Khimmed, Moscow, Russia) and Type I deionized water with a resistivity of 18.2 MΩ cm^−1^ were used to prepare the reaction mixtures, mobile phase for HPLC and stock solutions of mucolytic drugs.

Potassium permanganate (chem. pure), sodium hydroxide (chem. pure), hydrochloric acid (35–38%), sodium thiosulfate pentahydrate (for analysis), potassium iodide (chem. pure) and sulfuric acid (93.5–95.6%) purchased from Komponent-Reaktiv (Moscow, Russia) were used to obtain sodium hypochlorite from gaseous chlorine according to the known procedure [41], determine the active chlorine content [42] and terminate the reaction by quenching the active chlorine with thiosulfate-anions. According to the results of iodometric titration, the content of active chlorine in the prepared hypochlorite solution was 80 g L^−1^.

### 3.2. Model Chlorination of Bromhexine and Ambroxol

The procedures for both studied drugs were carried out under identical conditions. Stock solutions of BRO and AMB with an active substance concentration of 1000 mg L^−1^ were prepared by dissolving an accurately weighed portion of drug in methanol. Working solutions with a concentration of 10 mg L^−1^ were prepared by diluting the stock solution (100 μL) in 10 mL of 50 mM phosphate buffer solution at pH 7. The study of the transformation processes of AMB and BRO under aqueous chlorination conditions was carried out at three active chlorine concentrations in the reaction mixture: 0 (control sample), 4 and 7 mg L^−1^. The reaction was initiated by adding a required volume of sodium hypochlorite solution to the working solutions of drugs. After specified time intervals from the beginning of the reaction (1, 5, 10 and 60 min; 24 and 50 h), 0.5 mL of the mixture was taken, and 10 μL of 0.1 M sodium thiosulfate solution was immediately added to stop the reaction. The obtained samples were centrifuged and injected into the HPLC-HRMS system.

Similar working solutions (10 mg L^−1^ in 5 mL of 50 mM sodium phosphate at pH 7) were used to study the deep degradation products. The reaction was carried out in a 20 mL glass vial (for headspace analysis) with a screw cap and a PTFE septum. The required amount of sodium hypochlorite was added to achieve a significant excess of active chlorine (100 mg L^−1^). The reaction proceeded for 30 min and was then terminated by adding 400 μL of 0.1 M sodium thiosulfate solution. Then, ~2 g of sodium chloride was added to obtain a saturated solution, which was analyzed by GC-HRMS with headspace solid phase microextraction (HS-SPME).

### 3.3. Liquid Chromatography–High-Resolution Mass Spectrometry

HPLC-HRMS analysis of the reaction mixtures was carried out on a liquid chromatography–high-resolution mass spectrometry system consisting of an LC-30 Nexera liquid chromatograph (Shimadzu, Kyoto, Japan) and a TripleTOF 5600+ quadrupole time-of-flight mass spectrometer (AB Sciex, Concord, ON, Canada) equipped with a Duospray ion source and an automatic calibrant delivery system (CDS).

Chromatographic separation of the analytes was performed on a reversed-phase Zorbax Eclipse Plus C18 column, 150 × 3 mm, particle size 3.5 μm (Agilent Technologies, Santa Clara, CA, USA), in a gradient elution mode. The mixture of water (A) and methanol (B), both containing 0.1% formic acid, was used as a mobile phase with the following gradient program: 0–1 min: 15% B; 1–20 min: linear increase B to 100%; 20–30 min: 100% B. The column thermostat temperature was 40 °C, the mobile phase flow rate was 0.25 mL min^−1^, and the injection volume was 2 μL.

Mass spectrometry detection of analytes was carried out using electrospray ionization (ESI) in a positive ion mode. The ion source parameters were as follows: capillary voltage 5500 V; temperature 300 °C; curtain, drying and nebulizing gas pressure 30, 40 and 40 psi, respectively; declustering potential 60 V. The information-dependent acquisition (IDA) technique was used to obtain tandem (MS/MS) spectra. In this case, precursor ions with the minimum signal intensity of at least 100 cps were subjected to collision-induced dissociation (CID) with an applied collision energy (CE) of 40 eV with 20 eV spread. Mass spectra were recorded in the *m*/*z* ranges of 200–1000 (MS) and 20–1000 (MS/MS). Prior to each analysis, the mass scale was calibrated with sodium formate injected into the ion source by the CDS system.

The obtained MS data were processed using the PeakView version 2.2, MasterView version 2.0 and FormulaFinder version 2.2 software packages (AB Sciex, Concord, ON, Canada). The elemental compositions of the detected compounds were determined based on the following limits: C_0–100_, H_0–300_, O_0–20_, N_0–5_, Br_0–5_ and Cl_0–5_. The maximum allowed mass errors were <5 ppm (MS) and <10 ppm (MS/MS).

### 3.4. Gas Chromatography–High-Resolution Mass Spectrometry

Analysis of AMB and BRO deep degradation products via GC-HRMS was carried out using Pegasus GC-HRT+ 4D gas chromatography–high-resolution mass spectrometry system (LECO, St. Joseph, MI, USA) consisting of a folded flight path time-of-flight mass spectrometer, and an Agilent 7890 gas chromatograph (Agilent Technologies, Santa Clara, CA, USA) and an MPS robotic autosampler (Gerstel GmbH, Mülheim, Germany). Chromatographic separation was achieved on an Rxi-5MS capillary column (Restek, Bellefonte, PA, USA), 30 m × 0.25 mm, film thickness 0.25 μm. The oven program was as follows: initial temperature 30 °C, held for 3 min, linear ramp to 280 °C at a rate of 15 °C min^−1^, held at 280 °C for 4 min. The transfer line temperature was 280 °C. The carrier gas (He, 99.9999%, NII KM, Moscow, Russia) flow rate was 1.2 mL min^−1^.

Headspace solid-phase microextraction was used for concentrating the samples and injecting into the GC system. For this purpose, carboxen/divinylbenzene/polydimethylsiloxane (Car/DVB/PDMS) fiber (Sigma-Aldrich, Darmstadt, Germany) which ensures the sorption of the widest range of disinfection byproducts was used. The SPME procedure and extraction/desorption conditions typical for the analysis of various classes of DBPs [43] were applied. Headspace 20 mL glass vials with reaction mixtures were placed in an automatic agitator at a temperature of 50 °C, and sorption onto the fiber was carried out for 30 min. Desorption/injection was carried out at 250 °C for 2 min in splitless mode.

Mass spectrometric detection was performed in scanning mode (*m*/*z* 15–900) with a data-acquisition frequency of 40 Hz. Electron ionization mode (70 eV) was used. The ion source temperature was 200 °C. Instrument control, data acquisition and analysis were carried out using ChromaTOF software version 5.20. The NIST 2020 spectral library was used for the identification of the detected compounds.

## 4. Conclusions

In the processes of municipal wastewater disinfection with active chlorine, the widespread mucolytic drugs bromhexine and ambroxol undergo transformations to form a wide range of primary and final degradation products. At the initial stages, the reactions of cyclization with the formation of pyrazoline or tetrahydropyrimidine rings, radical hydroxylation and S_E_ chlorination of aromatic nuclei, *ipso*-substitution of bromine atoms with chlorine (*ipso*-S_E_) and oxidative N-dealkylation result in the occurrence of dozens DBPs, many of which were first discovered in the present study. Due to the presence of the reactive methyl group in the tertiary amine moiety, bromhexine differs from ambroxol in the greater variety of the primary transformation products. Deep degradation of both drugs leads to the formation of more than twenty volatile and semi-volatile compounds of six classes (trihalomethanes, dihaloacetonitriles, haloacetic acid esters, haloanilines, halobenzenes and haloindazoles), among which trihalomethanes predominate. Bromine-containing haloanilines are a specific class of degradation products of the studied drugs and include nine representatives, seven of which were not described earlier. One more discovered group of the novel DBPs associated with bromhexine and ambroxol is represented by halogenated indazoles formed through dealkylation of the primary transformation products containing pyrazoline or tetrahydropyrimidine cycle in their structure. The present study was limited to model experiments under simulated conditions. Thus, further research should be focused on an analysis of real wastewater samples and detailed characterization of the toxicity of the discovered DBPs. The obtained and anticipated results may contribute to the optimization and development of municipal wastewater-treatment technologies, providing more efficient removal of bromine-containing drugs and reducing their negative impact on aquatic ecosystems.

## Figures and Tables

**Table 1:** Disinfection byproducts detected at the early stages of bromhexine aqueous chlorination.

Compound	Retention Time, min	Elemental Composition	*m*/*z* [M + H]^+^	Error, ppm	*m*/*z* of Major Product Ions (Relative Intensity, %)
BRO	10.3	C_14_H_20_Br_2_N_2_	375.0067	0.3	261.8868 (100%); 292.8934 (43%); 263.8842 (28%)
B1	9.8	C_14_H_18_Br_2_N_2_	372.9910	−0.9	261.8868 (100%); 112.1131 (12%); 182.9677 (8%)
B2	13.7	C_14_H_17_Br_2_ClN_2_	406.9525	2.0	324.8740 (100%); 283.8472 (13%); 293.8312 (7%)
B3	9.6	C_14_H_18_BrClN_2_	329.0411	−1.1	217.9374 (100%); 112.1135 (13%); 246.9638 (4%)
B4	9.2	C_14_H_18_Cl_2_N_2_	285.0911	−3.4	173.9874 (100%); 112.1130 (24%); 203.0139 (4%)
B5	9.2	C_14_H_16_Br_2_N_2_	370.9745	−1.6	274.8945 (14%); 328.9291 (8%); 194.9676 (7%)
B6	8.9	C_14_H_16_Br_2_N_2_	327.0250	−2.5	230.9441 (19%); 284.9787 (12%); 151.0185 (3%)
B7	8.2	C_14_H_17_BrN_2_O	309.0591	−1.9	226.9814 (100%); 148.0633 (23%); 211.9575 (5%)
B8	9.1	C_14_H_17_ClN_2_O	265.1093	−3.1	183.0322 (100%); 148.0636 (15%); 168.0086 (5%)
B9	8.7	C_14_H_16_Br_2_N_2_O	386.9694	−2.1	311.9011 (9%); 337.9181 (3%); 110.0974 (3%)
B10	8.5	C_14_H_16_BrClN_2_O	343.0207	−1.0	260.9426 (100%); 182.0239 (33%); 181.0159 (10%)
B11	6.6	C_14_H_21_BrN_2_O	313.0903	−2.2	199.9711 (100%); 114.1287 (15%); 171.9711 (4%)
B12	6.2	C_14_H_21_ClN_2_O	269.1418	1.0	156.0208 (100%); 114.1283 (50%); 128.0255 (8%)
B13	10.2	C_13_H_18_Br_2_N_2_	360.9898	−3.5	261.8860 (100%); 182.9675 (8%); 104.0504 (2%)
B14	9.8	C_13_H_18_BrClN_2_	317.0405	−2.7	217.9366 (100%); 139.0185 (5%)
B15 (AMB)	7.2	C_13_H_18_Br_2_N_2_O	376.9860	1.4	261.8874 (100%); 182.9678 (6%)
B16	6.1	C_7_H_5_Br_2_N	261.8849	−3.2	182.9662 (35%); 181.9584 (19%); 155.9565 (15%)
B17	6.4	C_7_H_5_BrClN	217.9367	−0.8	104.0512 (30%); 139.0184 (22%); 138.0118 (17%)
B18	9.2	C_7_H_5_Cl_2_N	173.9870	−0.5	- *
B19	6.8	C_8_H_10_Br_2_N_2_	292.9273	−3.2	261.8863 (100%); 263.8842 (25%); 182.9677 (16%); 194.9674 (10%)
B20	15.7	C_8_H_6_Br_2_N_2_	288.8967	1.2	209.9786 (100%); 208.9712 (70%); 273.8735 (5%)
B21	9.2	C_8_H_7_BrN_2_O	226.9815	−0.2	148.0615 (100%); 147.0552 (65%); 211.9584 (50%)

* Low signal intensity of the precursor ion.

**Table 2:** Disinfection byproducts detected in the early stages of the interaction of ambroxol with active chlorine.

Compound	Retention Time, min	Elemental Composition	*m*/*z* [M + H]^+^	Error, ppm	*m*/*z* of Major Product Ions (Relative Intensity, %)
AMB	7.2	C_13_H_18_Br_2_N_2_O	376.9866	2.0	261.8874 (100%); 182.9678 (6%)
A1	6.7	C_13_H_18_BrClN_2_O	333.0364	−0.1	217.9364 (100%); 230.9931 (1%); 194.9721 (1%)
A2	3.0	C_13_H_19_BrN_2_O_2_	315.0701	0.4	199.9702 (100%); 171.9758 (4%)
A3	16.1	C_13_H_14_Br_2_N_2_O	372.9553	2.0	261.8850 (30%); 288.8955 (10%); 194.9669 (6%)
A4	16.2	C_13_H_14_Br_2_N_2_O_2_	388.9495	0.6	290.8758 (100%); 211.9571 (12%); 370.9368 (1%)
A5	6.1	C_7_H_5_Br_2_N	261.8860	−0.6	182.9670 (30%); 181.9594 (12%); 104.0503 (11%)
A6	5.5	C_7_H_5_BrClN	217.9363	−1.7	139.0183 (35%); 104.0503 (19%); 112.0060 (11%)

**Table 3:** Deep transformation products of ambroxol and bromhexine.

N	Retention Time, s	Elemental Composition	Assumed Compound	% of the Total Peak Area of the Detected DBPs	
				BRO	AMB
THMs					
1	176	CHCl_3_	Trichloromethane	2.5	3.7
2	267	CHBrCl_2_	Bromodichloromethane	22.6	10.7
3	381	CHBr_2_Cl	Dibromochloromethane	54.3	64.5
4	495	CHBr_3_	Tribromomethane	12.0	12.4
DHANs					
5	283	C_2_HCl_2_N	Dichloroacetonitrile	0.1	0.3
6	407	C_2_HBrClN	Bromochloroacetonitrile	0.2	1.3
7	527	C_2_HBr_2_N	Dibromoacetonitrile	0.1	0.8
HAEs					
8	442	C_3_H_4_Cl_2_O_2_	Methyl ester dichloroacetic acid	0.03	0.1
9	539	C_3_H_4_BrClO_2_	Methyl ester bromochloroacetic acid	0.1	1.1
10	627	C_3_H_4_Br_2_O_2_	Methyl ester dibromoacetic acid	0.1	2.0
Haloanilines					
11	1000	C_6_H_4_Cl_3_N	2,4,6-trichloroaniline	2.7	0.1
12	1073	C_6_H_4_BrCl_2_N	4-Bromo-2,6-dichloroaniline	0.8	-
13	1077	C_6_H_4_BrCl_2_N	2-Bromo-4,6-dichloroaniline	0.6	0.2
14	1144	C_6_H_4_Br_2_ClN	2,6-Dibromo-4-chloroaniline	0.2	0.01
15	1148	C_6_H_4_Br_2_ClN	2,4-Dibromo-6-chloroaniline	1.2	0.5
16	1713	C_7_H_4_Br_2_ClNO	2,4-Dibromo-6-chloro-2-aminobenzaldehyde	0.02	0.1
17	1217	C_6_H_4_Br_3_N	2,4,6-tribromoaniline	0.6	0.1
18	1123	C_6_H_5_BrClN	4-bromo-2-chloroaniline	0.1	0.3
19	1195	C_6_H_5_Br_2_N	2,4-dibromoaniline	1.3	0.1
20	1118	C_7_H_7_BrClNO	3-Bromo-5-chloro-2-methoxyaniline	0.2	0.8
21	1185	C_7_H_7_Br_2_NO	2,6-dibromo-4-methoxyaniline	-	0.5
Halobenzenes					
22	933	C_6_H_3_Br_2_Cl	1,3-dibromo-5-chlorobenzene	0.04	0.1
Haloindazoles					
23	1318	C_8_H_6_BrClN_2_	5-Bromo-7-chloro-2-methylindazole	0.1	0.1
24	1383	C_8_H_6_Br_2_N_2_	5,7-Dibromo-2-methylindazole	0.1	0.3

## Data Availability

The data presented in this study are available in the article.
